# Supplementary material for: 64Cu-ATSM Predicts Efficacy of Carbon Ion Radiotherapy Associated with Cellular Antioxidant Capacity
Source: Cancers (Basel). 2021 Dec 7;13(24):6159. doi: 10.3390/cancers13246159 (PMC8699283; doi:10.3390/cancers13246159)

Supplementary Figure S1

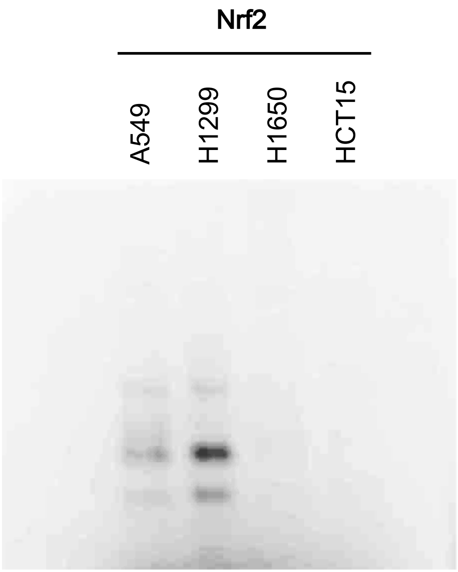

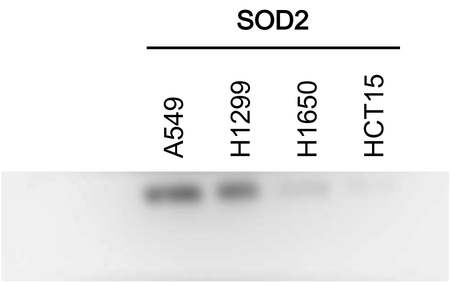

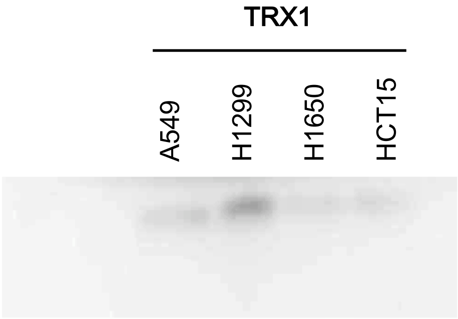

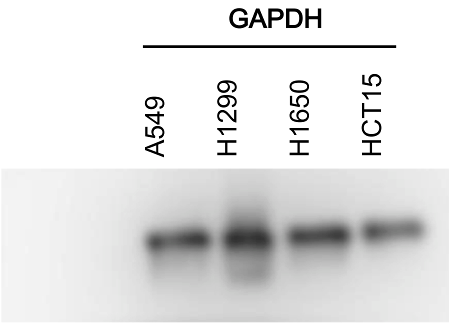

Supplementary Figure S2

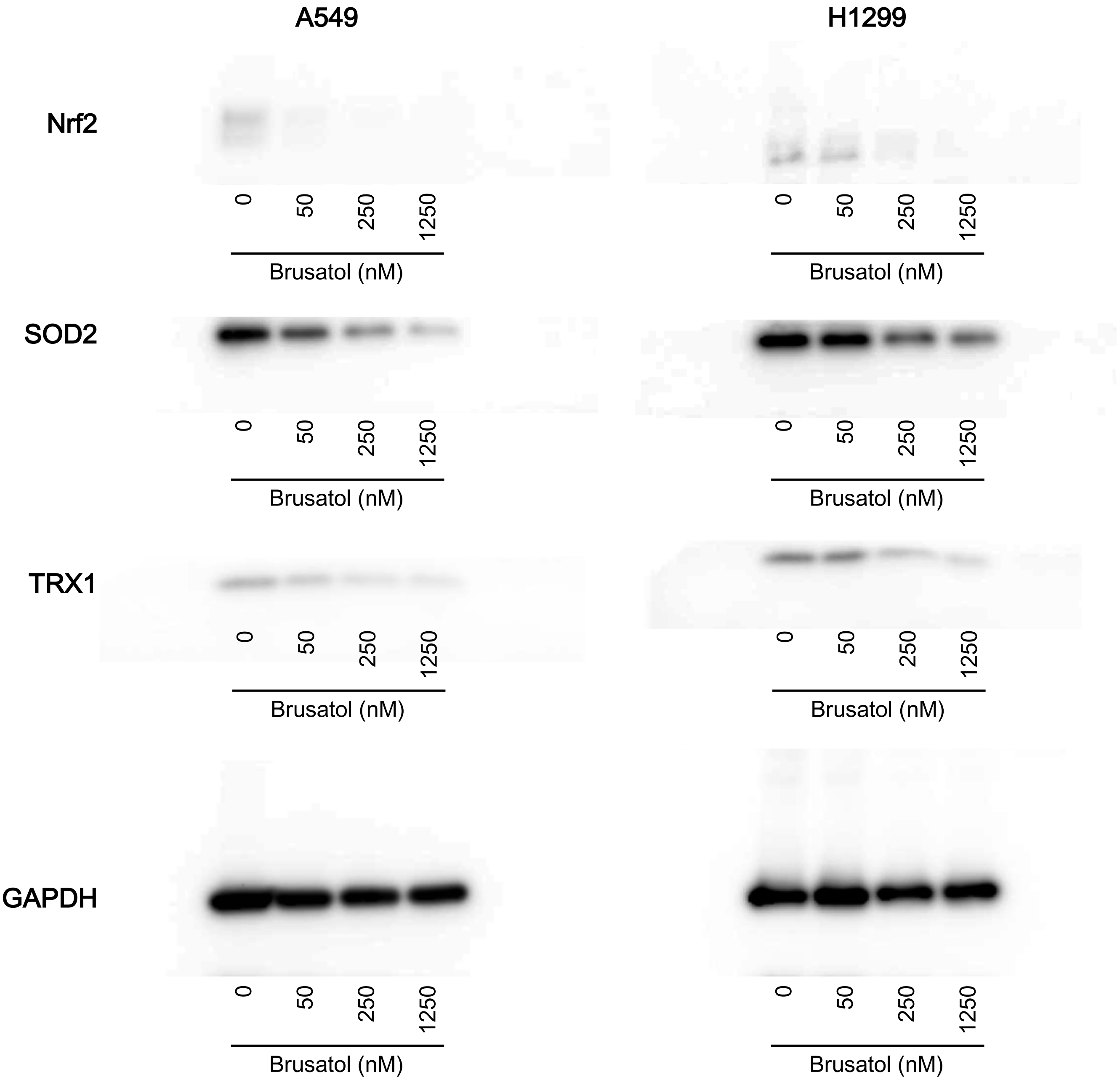

Supplementary Figure S3

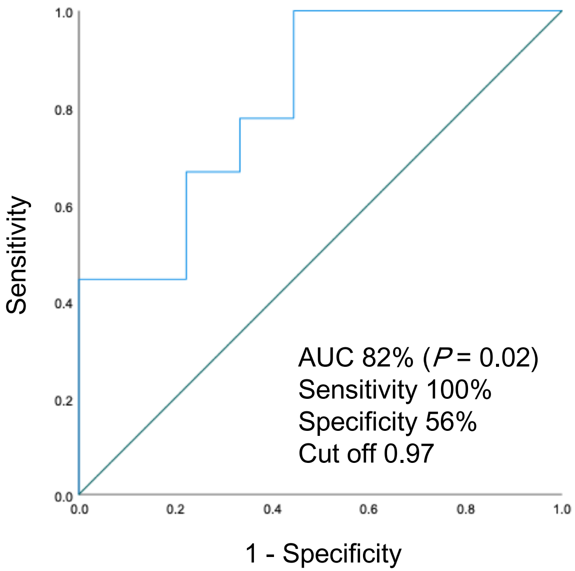

Supplementary Figure S4

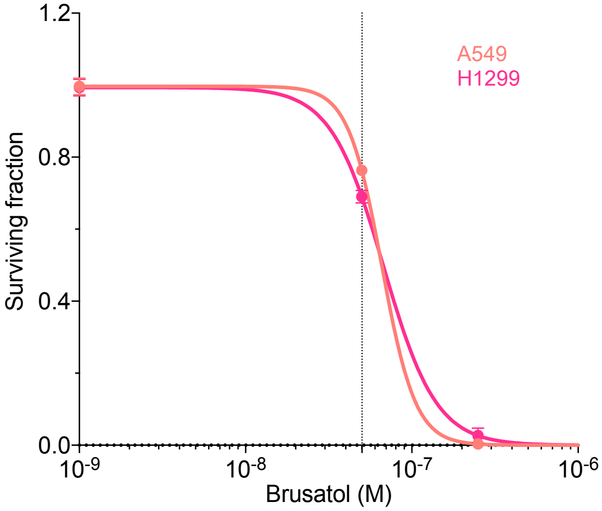

Supplement: Supplementary file 1 [file cancers-13-06159-s001.zip › Supplementary Figures.pdf]
