# Supplementary material for: 64Cu-ATSM Predicts Efficacy of Carbon Ion Radiotherapy Associated with Cellular Antioxidant Capacity
Source: Cancers (Basel). 2021 Dec 7;13(24):6159. doi: 10.3390/cancers13246159 (PMC8699283; doi:10.3390/cancers13246159)
Supplement: Supplementary file 1 [file cancers-13-06159-s001.zip › Supplementary Tables.pdf]

Supplementary Table S1. Origin and histology of cancer cell lines used in this study.

**Cell line**	**Origin**	**Histology**
FaDu	Head and neck	Squamous cell carcinoma
H1703	Lung	Squamous cell carcinoma
A549	Lung	Adenocarcinoma
PC-9	Lung	Adenocarcinoma
Ma24	Lung	Adenocarcinoma
H1650	Lung	Adenocarcinoma
H1299	Lung	Large cell carcinoma
HCT15	Colon	Adenocarcinoma
PC-3	Prostate	Adenocarcinoma
U2OS	Tibia	Osteosarcoma

Supplementary Table S2. Antibodies used in this study.

Target	Species	Clonality	Dilution rate	Company	Product#
Nrf2	Rabbit	Monoclonal	1:200	Abcam	ab62352
SOD2	Rabbit	Monoclonal	1:1000	Cell Signaling Technology	13194
TRX1	Rabbit	Polyclonal	1:1000	Cell Signaling Technology	2285
GAPDH	Rabbit	Monoclonal	1:1000	Cell Signaling Technology	3683
